# Magnetic resonance imaging of the pulsing brain: a systematic review

**DOI:** 10.1007/s10334-022-01043-1

**Published:** 2022-10-15

**Authors:** Alanoud Almudayni, Meshal Alharbi, Alimul Chowdhury, Jonathan Ince, Fatmah Alablani, Jatinder Singh Minhas, Andrea Lecchini-Visintini, Emma Ming Lin Chung

**Affiliations:** 1 College of Applied Medical Sciences, Prince Sattam Bin Abdulaziz University, Al-Kharj, Saudi Arabia; 2 Cerebral Haemodynamics in Ageing and Stroke Medicine (CHiASM) Research Group, Department of Cardiovascular Sciences, University of Leicester, Room 419, Robert Kilpatrick Building, Leicester Royal Infirmary, Infirmary Square, Leicester, LE1 5WW UK; 3 College of Applied Medical Sciences, King Saud Bin Abdulaziz University for Health Sciences, Riyadh, Saudi Arabia; 4 University Hospitals of Leicester NHS Trust, Leicester, LE1 5WW UK; 5 National Institute for Health Research Leicester Biomedical Research Centre, University of Leicester, Leicester, LE5 4PW UK; 6 School of Electronics and Computer Science, University of Southampton, Southampton, SO17 1BJ UK; 7 School of Life Course and Population Sciences, King’s College London, Room 3.25a, Shepherd’s House, Guy’s Campus, King’s College London, London, SE1 7EH UK

**Keywords:** Brain tissue displacement, Brain tissue pulsation, Magnetic resonance imaging, MRI

## Abstract

**Objective:**

To perform a systematic review of the literature exploring magnetic resonance imaging (MRI) methods for measuring natural brain tissue pulsations (BTPs) in humans.

**Methods:**

A prospective systematic search of MEDLINE, SCOPUS and OpenGrey databases was conducted by two independent reviewers using a pre-determined strategy. The search focused on identifying reported measurements of naturally occurring BTP motion in humans. Studies involving non-human participants, MRI in combination with other modalities, MRI during invasive procedures and MRI studies involving externally applied tests were excluded. Data from the retrieved records were combined to create Forest plots comparing brain tissue displacement between Chiari-malformation type 1 (CM-I) patients and healthy controls using an independent samples *t*-test.

**Results:**

The search retrieved 22 eligible articles. Articles described 5 main MRI techniques for visualisation or quantification of intrinsic brain motion. MRI techniques generally agreed that the amplitude of BTPs varies regionally from 0.04 mm to ~ 0.80 mm, with larger tissue displacements occurring closer to the centre and base of the brain compared to peripheral regions. Studies of brain pathology using MRI BTP measurements are currently limited to tumour characterisation, idiopathic intracranial hypertension (IIH), and CM-I. A pooled analysis confirmed that displacement of tissue in the cerebellar tonsillar region of CM-I patients was + 0.31 mm [95% CI 0.23, 0.38, *p* < 0.0001] higher than in healthy controls.

**Discussion:**

MRI techniques used for measurements of brain motion are at an early stage of development with high heterogeneity across the methods used. Further work is required to provide normative data to support systematic BTPs characterisation in health and disease.

**Supplementary Information:**

The online version contains supplementary material available at 10.1007/s10334-022-01043-1.

## Introduction

Brain tissue motion can be measured non-invasively using MRI, but the clinical value of such measurements has yet to be established. It has long been known that the healthy brain pulsates with each cardiac cycle, where the brain slightly expands and distorts in shape to maintain a balance between compartmental pressures and changes in arterial blood volume [1]. These repetitive intrinsic brain tissue pulsations (BTPs) are sensitive to both cardio- and cerebrovascular physiology, as well as the biomechanical properties of the brain, blood and cerebral spinal fluid (CSF) compartments. It can be hypothesised that brain pathology affects regional BTPs [2, 3], providing a potential ‘window on the brain’ for diagnosing brain pathology [4, 5].

Existing literature has described the measurement of BTPs using both MRI [6–8] and ultrasound [2, 9–11]. A previous systematic review by Ince et al*.* focused on the use of ultrasound for measuring BTPs and hypothesised there may be an effect of cerebral pathophysiology on brain motion [2]. Since then, additional literature has allowed further exploration of the effect of pathology on brain motion, including a healthy volunteer study of 107 participants [12] and a study measuring BTP transcranial tissue Doppler (TCTD) measurements in acute stroke.

These later studies used transcranial tissue Doppler (TCTD), which due to the high temporal resolution of Doppler ultrasound measurements makes this technique particularly suitable for physiological measurement studies [13].

Studies investigating measurement of BTP motion using MRI mostly predate the use of ultrasound methods and predominantly focus on quantifying motion in specific brain regions. Given the increasing interest in BTP measurement techniques across both ultrasound and MRI, a summary of existing MR measurement techniques appears timely and may be useful for guiding future research.

This systematic review looks to provide an overview of available MRI methods for investigating cardiac-induced brain tissue motion in humans, highlighting key findings and suggestions for future work.

## Materials and methods

A systematic search was conducted to identify all studies in humans that had used MRI methods to measure naturally occurring brain tissue motion. Retrieved records were reviewed and summarised using a narrative synthesis and reported in accordance with the Preferred Reporting Items for Systematic Reviews and Meta-Analysis (PRISMA) [14] guidelines.

### Search strategy

Our systematic review protocol was prospectively designed and registered with the PROSPERO database (Registration number: CRD42019158288). A systematic database search of MEDLINE OVID (1946–current), SCOPUS (1966–current), and OpenGrey were conducted using a pre-agreed protocol by 2 reviewers (AA and MA). Searches took place on November 2019 and May 2021 (to identify any further publications). Search terms included: (Brain OR Cerebral OR Neuro) AND (Tissue*) AND (Displace* OR Puls* OR Move* OR Motion OR Amplitude) AND (Magnetic Resonance Imaging OR MRI OR MR). The search was limited to original peer-reviewed research, published in English, with no limits placed on publication date. A filter was applied to restrict the results to studies conducted in humans.

### Eligibility criteria

After discarding duplicate publications, titles and abstracts were reviewed against pre-defined eligibility criteria by 2 reviewers (AA and MA). All records reporting MR techniques in combination with brain motion measurements were eligible for full-text review. Independent full-text review by the same reviewers was then used to identify papers specifically focusing on measurement of naturally occurring endogenous brain tissue motion in humans. Studies focusing exclusively on brain tissue elastography or analysis of brain motion in response to external stimuli were excluded. Records were also excluded for the following reasons: studies using phantoms or non-human participants, studies using MRI in combination with other modalities, studies using MRI during invasive procedures and studies involving externally applied test (paradigms) or stimulation (e.g. vibrations or ultrasound) to induce motion of brain tissue. Exclusion criteria included conference abstracts and articles without full-text access.

Eligible records then underwent independent full-text review by AA, MA and AC, including review of citations and references to identify any additional relevant articles meeting the search eligibility criteria.

### Data extraction

All eligible full-text articles were independently assessed by two researchers (AA and MA) to extract the following information from the reports: (i) names of the authors, (ii) publication date, (iii) study design, (iv) participant condition (e.g. control or pathology), (v) number of subjects, (vi) age, (vii) sex, (viii) main MRI acquisition methods, including sequence type and imaging planes and (ix) tissue displacement or motion measurement general observations (Supplementary Appendix 1). MRI aspects of each paper were reviewed by an MR physicist with experience in brain imaging (AC).

### Quality assessment

The quality of included records was determined independently by two reviewers (AA and MA) using a pre-defined quality assessment tool (Supplementary Appendix 2) [15]. This included 15 points for relevant items, each of which were equally weighted and scored to evaluate the methodological quality and completeness of reports for the purposes of our review. Any disagreement in quality assessment was resolved through discussion and consensus by the two main reviewers (AA and MA).

### Statistical analysis

Data from the retrieved records were combined to create Forest plots comparing brain tissue displacement between CM-I patients and healthy controls. Distributions were assessed for normality using a Shapiro–Wilk test, with normally distributed data presented as a mean (plus standard deviation) and non-normally distributed data summarised by the median and interquartile range (IQR). Comparison between CM-I patients and control subjects was performed using an independent samples *t*-test (two-tailed). A *p*-value of < 0.05 was considered statistically significant.

### Literature search results

**Fig. 1:**
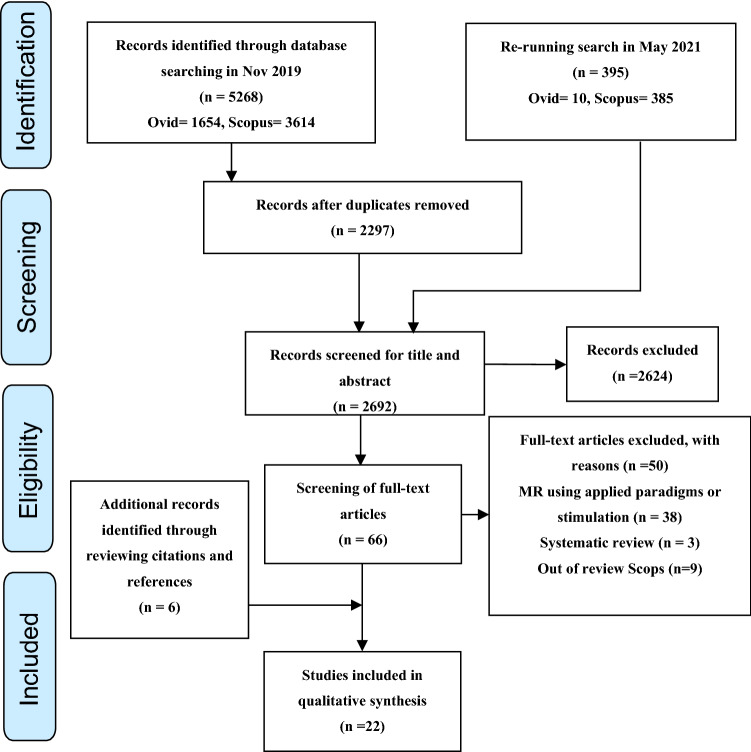
Modified PRISMA flowchart summarising the systematic search process [14]

The stated search strategy initially yielded 5268 records from SCOPUS and MEDLINE databases. No records were retrieved from the OpenGrey database. A further 395 records were identified after a repeat search in May 2021. After removing duplicate entries, screening of abstracts and titles and screening the full text of 66 papers, an additional 6 records were identified based on citations and references. Including these 6 papers, a total of 22 papers fitted our inclusion criteria, limited to the application of MRI to natural (endogenous) brain tissue pulsations. A modified version of the PRISMA flowchart, summarising the processes for systematic identification of eligible papers, is provided in Fig. 1 [14].

### Quality assessment

The quality assessment scores of papers included for full-text review showed studies were of variable quality, with scores ranging from a minimum of 7/15 to maximum of 14/15, Supplementary Appendix 3. Points were mainly lost due to unclear descriptions of the methods used, and uncertain inclusion and exclusion criteria and demographics of participants. As studies were largely exploratory, sample sizes were typically not considered prior to data gathering and it was therefore difficult to establish whether any comparisons made between studies were satisfactorily powered.

The two reviewers noted significant heterogeneity in methods, study design and the analysis and presentation of findings. Heterogeneity existed in the methods for acquiring the brain tissue pulsatility data, as well as analysis techniques for establishing and reporting the brain tissue displacement and motion. Studies were performed using scanners from different manufacturers (including Philips, Siemens and GE) and at various field strengths: 1 T, 1.5 T, 3 T and 7 T. Different types of imaging sequences were used for contrast and spatial encoding, including adaptation of spin echo, gradient echo, echo-planar imaging (EPI), Spiral and balanced steady-state free precession (bSSFP) techniques. Image acquisitions were either 2D or 3D, using either prospective or retrospective cardiac-gating (electrocardiogram (ECG) or finger plethysmography). Tissue motion results were expressed as 1D, 2D or 3D velocity or displacement vectors, with different views including axial, sagittal and coronal imaging planes. Therefore, there was significant heterogeneity in the reported methods for BTP measurements. A narrative review summarising the available outcome data was therefore performed. All statistical analyses were conducted using Prism GraphPad (GraphPad Software, San Diego, USA).

### Characteristics of the reported studies

The main study characteristics of the included publications are summarised in Supplementary Appendix 1. Study populations varied in size (ranging from 2 to 89 participants). A total of 286 subjects were studied across all 22 publications [198 healthy /control subjects, 3 brain tumour patients, 3 Idiopathic Intracranial Hypertension (IIH) patients and 76 Chiari malformation (CM) patients]. As detailed in Supplementary Appendix 1, some studies did not describe the age or sex of participants.

### MRI methods identified

The retrieved records were reviewed in chronological order to develop an impression of the development of BTP measurement methods over time. MRI methods for visualising and quantifying brain tissue motion use cardiac-gated MRI techniques that can be classified into 5 main methods: (i) Phase-contrast MRI [6–8, 16–19], (ii) Complementary Spatial Modulation of Magnetization (CSPAMM) [20], (iii) Displacement Encoding with Stimulated Echoes (DENSE) [3, 4, 21–26], (iv) anatomical landmark motion tracking, to measure displacements in images acquired with cardiac-gated cine balanced steady-state gradient echo sequences through evaluation of pixel-shifting [27, 28], (v) and amplified MRI (aMRI) [5, 29–31].

General differences between measurement principles are; (i) phase-contrast MRI uses bipolar gradients to encode movement as a phase change in complex MRI images and estimate tissue velocity over the cardiac cycle [6–8, 16–19], (ii) CSPAMM or tagging sequences are based on separating the component of the magnetization with the tagging information from the relaxed component by subtraction of two opposite direction measurements to remove untagged signal and then using harmonic phase (HARP) post-processing to extract the harmonic peak containing all motion information [20], (iii) DENSE MRI is based on encoding tissue displacements to record the phase of the stimulated echo signal over time. Displacements in all three spatial directions can be measured using a cardiac-gated cine, with 2D or 3D segmented echo-planar imaging (EPI) readout [3, 4, 21–26], (iv) evaluation of “pixel-shifting” is based on tracking anatomical landmarks within the images over time. Images are acquired using a high temporal resolution cine MR sequence, such as balanced steady-state free precession (bSSFP) (including FIESTA, bFFE) or echo-planar imaging (EPI) [27, 28] and (v) amplified MRI (aMRI) retrospectively amplifies tissue movements seen in cine movies acquired with bSSFP sequence, using an Eulerian video magnification (EVM), or phase-based (3D aMRI) algorithm to allow qualitative assessment of motion [5, 29–31]. The general advantages and disadvantages of these MRI methods are summarised in Table 1.

**Table 1:** General advantages and disadvantages of each MRI method

Methods	Advantages	Disadvantages
Phase-contrast MRI [6–8, 16–19]	Allows quantitative velocity measurements Good motion specificity Easy to apply in conventional MRI	Indirect measurement of displacement Sensitive to phase errors arising from eddy currents and gradient non-linearities Requiring numerical integration steps to quantify displacement data Requires multiple velocity encoding gradient directions and values to capture motion in all relevant directions, which can lead to long scan times Limited signal-to-noise ratio (SNR)
Complementary Spatial Modulation of Magnetization (CSPAMM) [20]	Quantitative method to measure motion in term of displacement Allows direct quantification of periodic caudal brain tissue displacement with no need to numerical integration steps Insensitive to phase-related image artefacts	Limited SNR Limited displacement sensitivity and spatial resolution when measuring very small displacements Limited to only quantify brain motion measurement in the cranial–caudal direction Reduced reproducibility of displacement measurements occurs at later cardiac phases because of tag fading caused by T1 relaxation decay Spatial blurring of the displacement in edges of structures moving independently
Displacement Encoding with Stimulated Echoes (DENSE) [3, 4, 21–26]	Quantitative method to measure motion in terms of displacement and strains Direct measurement of the displacement High spatial and temporal resolution Sensitive to very small (0.01 mm) brain displacements with good reproducibility	Long scan time Causes ghosting artefacts Limited SNR Limited accuracy in dynamic displacement
Anatomical landmark motion tracking using pixel-shifting analysis [27, 28]	Quantitative method to measure motion in term of velocity and displacement Good for analysis neural structure within the intracranial CSF Sensitive to detect cephalad and caudad cerebellar tonsil motion	Long scan time Less accuracy and precision for measuring soft-tissue displacements Accuracy of timing parameter may be affected if lengths of trigger parameter delay Not very sensitive to sub-voxel motion
Amplified MRI (aMRI) [5, 29–31]	Qualitative method to visualise displacement Short scan time compared to phase-contrast MRI Ability to reveal smaller motions Does not require phase-encoding in multiple directions to capture the full extent of brain motion Can achieve higher spatial resolution compared to DENSE High SNR Easy to implement using existing scan sequences	Currently lacks the ability to directly quantify motion

### Motion of healthy brain tissue

Data acquired using different MRI techniques (phase-contrast MRI, CSPAMM, DENSE and aMRI) from a total of 164 healthy subjects in studies carried out between 1987 and 2021 were collated to provide a better overall understanding of healthy brain tissue motion.

Our current picture of brain tissue motion over the cardiac cycle is as follows; in early systole, blood flow entering the brain leads to expansion of the major cerebral arteries and an increase in brain blood volume. As the cranium cannot expand to accommodate this extra volume the cerebral compartments increase in pressure resulting in increased intracranial pressure (ICP), and subsequent adjustment of intracranial pressures and volumes according to the Monro–Kellie doctrine. The resultant motion of the brain appears to involve rapid caudal motion of the brainstem and displacement of CSF through the foramen magnum down into the spinal subarachnoid space. In diastole, there is a gradual return of brain tissue to a neutral position and the CSF then moves cephalically, back towards the brain [7, 8, 16, 20, 21].

Despite using different techniques, all studies agree that maximum tissue displacement occurs during systole. Overall, brain motion was found to be strongest in the cephalocaudal direction [8], followed by lateral motion, with relatively little movement in the anteroposterior direction [7, 20].

**Fig. 2:**
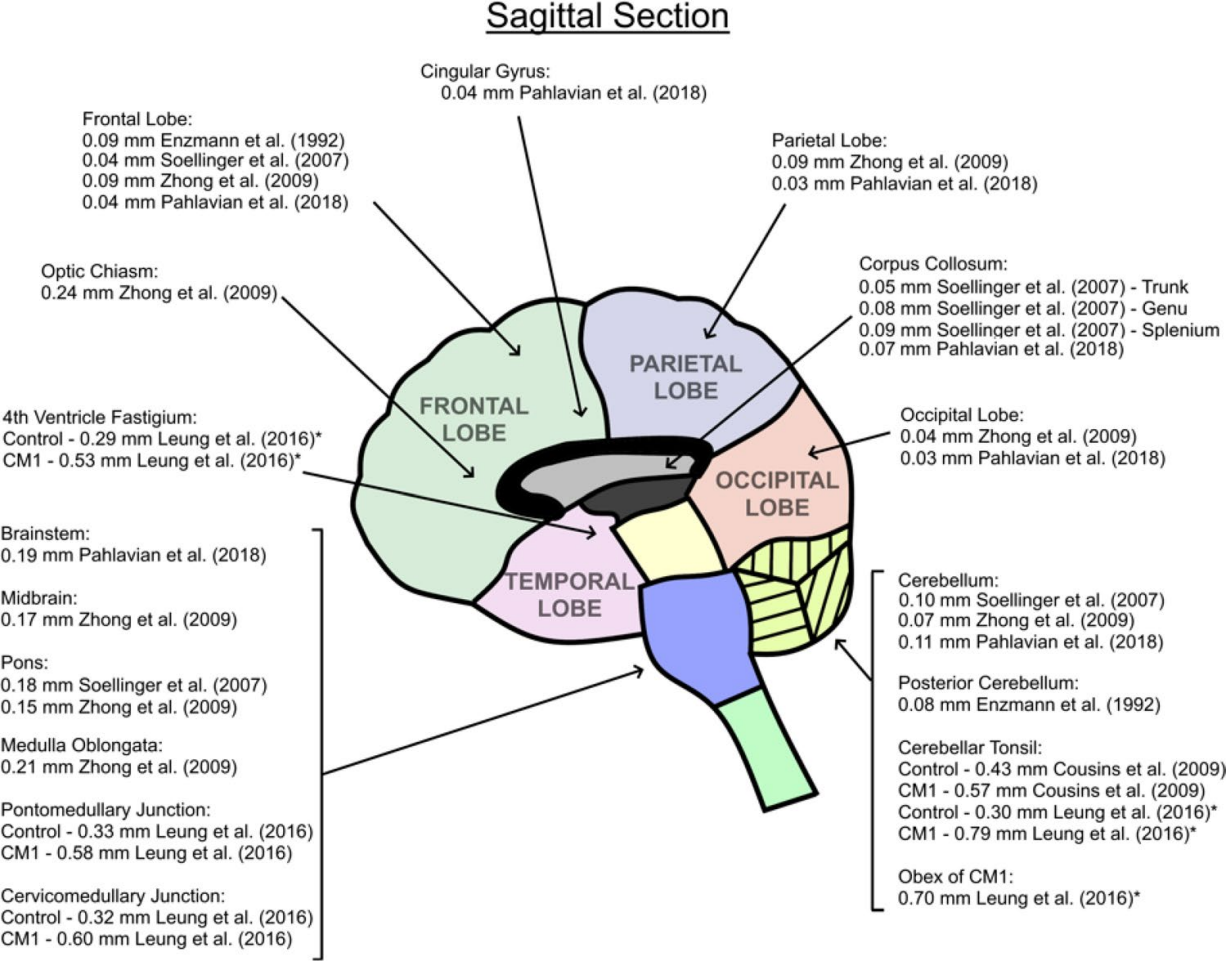
Reported tissue displacement estimates for differing brain regions measured in sagittal section

**Fig. 3:**
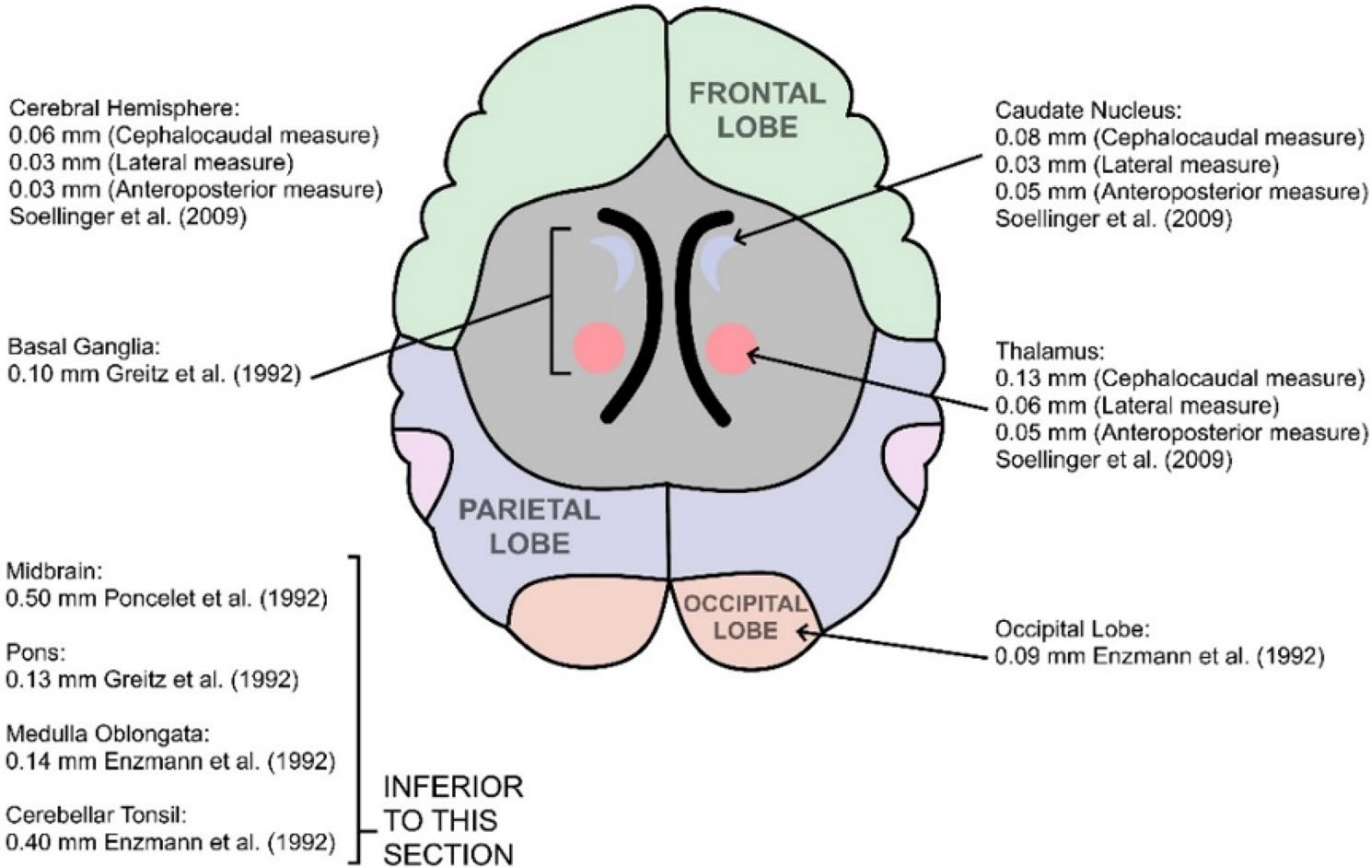
Reported tissue displacement estimates for differing brain regions measured in axial section

**Fig. 4:**
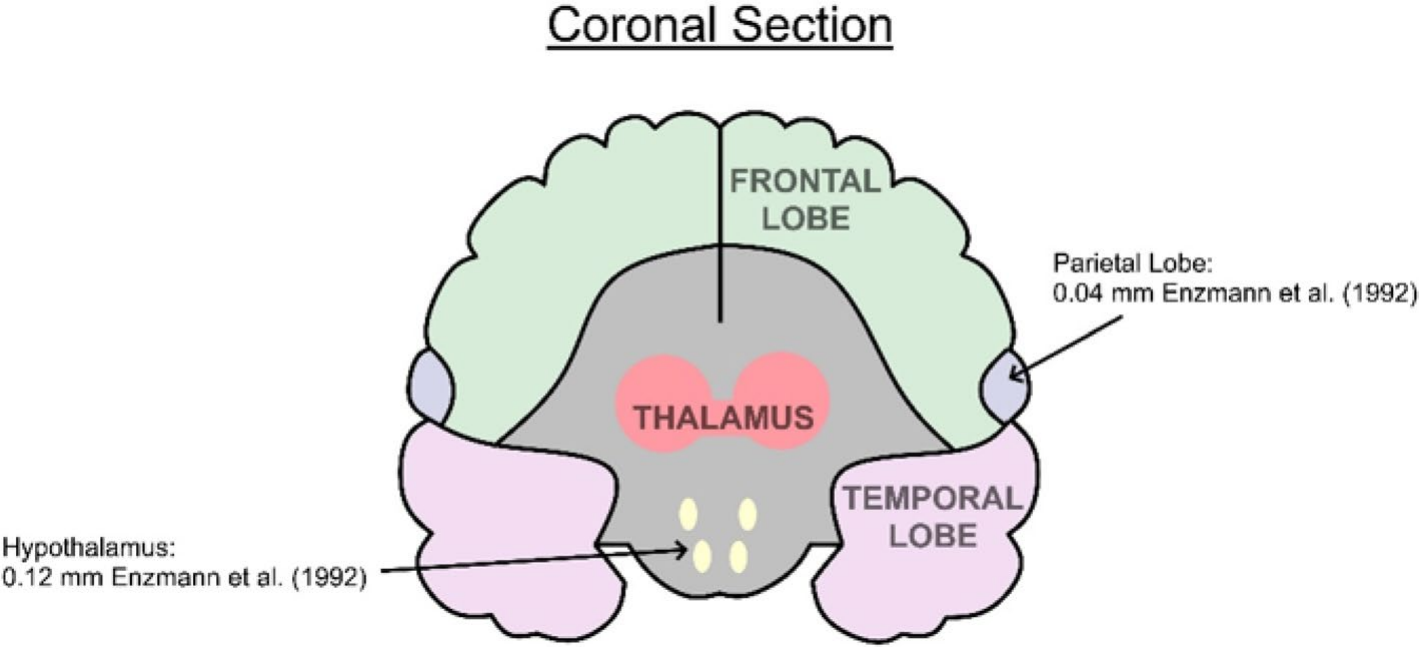
Reported tissue displacement estimates for differing brain regions measured in coronal section

All studies reported the largest velocities and displacements of the brain occurred close to the central brain regions and base of the brain, in tissue adjacent to the circle of Willis, foramen magnum and brain stem (midbrain, pons and medulla). Six studies reported motion in terms of velocity estimates [6–8, 16, 17, 27 (Supplementary Appendix 4). The majority of studies reported displacements (or both) [3, 4, 6–8, 21, 27, 28, 31]. A summary of reported displacements in the sagittal (Fig. 2), axial (Fig. 3) and coronal planes (Fig. 4) is provided in Figs. 2, 3, 4. A handful of studies reported motion in terms of tissue strain, where peak strain was reported in the basal ganglia [24–26, 32]. The reason for variations in the choice of measurement outcomes across studies is unclear. Some studies provided qualitative or quantitative estimates describing the timing of peak displacement [3, 4, 6–8, 18, 21, 27, 28, 31], confirming that central and basal tissue structures reach peak displacement earlier than peripheral brain regions. Zhong et al*.* [21] found central brain tissue structures reach peak displacement approximately 250– 300 ms after the start of the R–R interval compared to 450–500 ms for the peripheral brain lobes.

Six studies involving a total of 55 healthy subjects compared motion of all four lobes of the brain (frontal, parietal, temporal and occipital) using phase-contrast or DENSE MR. The occipital lobe is reported as having the smallest motion, while the parietal lobe has the largest [3, 4, 7, 8, 21, 24]. Modelling of regional brain motion could be the subject of future investigation.

Four studies involving a total of 31 healthy participants compared strain within grey matter (GM) and white matter (WM) regions using phase-contrast [18], or DENSE [24–26] MR. These studies suggest a significant difference between the GM and WM, with higher volumetric strains observed in WM regions using DENSE MR [24–26].

### Motion of brain tissue in disease

MRI shows great promise as a non-invasive method for assessing the mechanical properties and movement of brain tissue in the presence of the following pathologies:

#### Tumour

A study by Wirestam et al. compared brain tissue motion in 3 tumour patients (2 patients with astrocytoma and one patient with meningioma) with data from 8 healthy volunteers, using phase-contrast MRI. Maximum velocities in the central parts (thalamus) of the brain were generally lower in the patient with meningioma (0.3 mm/s) compared to healthy subjects (0.94 mm/s). No difference in maximum velocities was observed between the patient with astrocytoma (0.7–1.1 mm/s) and healthy controls (0.5–1.5 mm/s) [17]. Based on these studies, phase-contrast MRI appears to be a suitable method for sensitively measuring brain tissue motion, with the added advantage of being easy to implement using conventional MR scanners, allowing the utilisation of this method in clinical or research settings. Phase-contrast MRI methods were utilised mainly in earlier studies with a view to explaining CSF motion [6, 16]. However, long scan times, relatively low SNR and poor accuracy in dynamic displacement derivation, representative limitations that would need to be addressed. See Table 1 for a summary of the strengths and limitations of each method.

#### Idiopathic Intracranial Hypertension (IIH)

A study by Saindane et al*.* measured brain tissue motion in 9 IIH patients compared to 9 healthy volunteers using the DENSE MRI method. This study found that patients with IIH had lower brain pontine motion (0.06 mm) than control subjects (0.11 mm) [22]. This study also assessed correlation between ICP status and brain motion in IIH patients; ICP was found to be elevated in IIH patients. The study found that after reducing ICP in IIH patients through CSF removal via a lumbar puncture, pontine displacement for all patients with IIH increased to normal values with mean pontine displacement similar to that of healthy control subjects [22]. This study suggests that there may be a correlation between ICP and brain motion, with high ICP constraining brain motion, which can be normalised by reducing ICP through the removal of CSF.

DENSE MRI appears to be suitable for detecting small changes in brain motion and can be used for directly measuring brain displacements with high spatial and temporal resolution. Further development of this method to improve signal-to-noise ratio, reproducibility and accuracy of displacement measurements will be beneficial.

#### Chiari malformation (CM-I)

Three studies compared displacement of specific brain regions in patients and/or healthy controls using a “pixel-shifting” method to track motion of anatomical landmarks in images acquired using cardiac-gated cine balanced steady-state gradient echo sequences (sagittal plane 2D FIESTA or bFFE) [8, 27, 28]. One study used phase-based aMRI to qualitatively compare brain motion in one CM-I patient to one control subject [5].

**Fig. 5:**
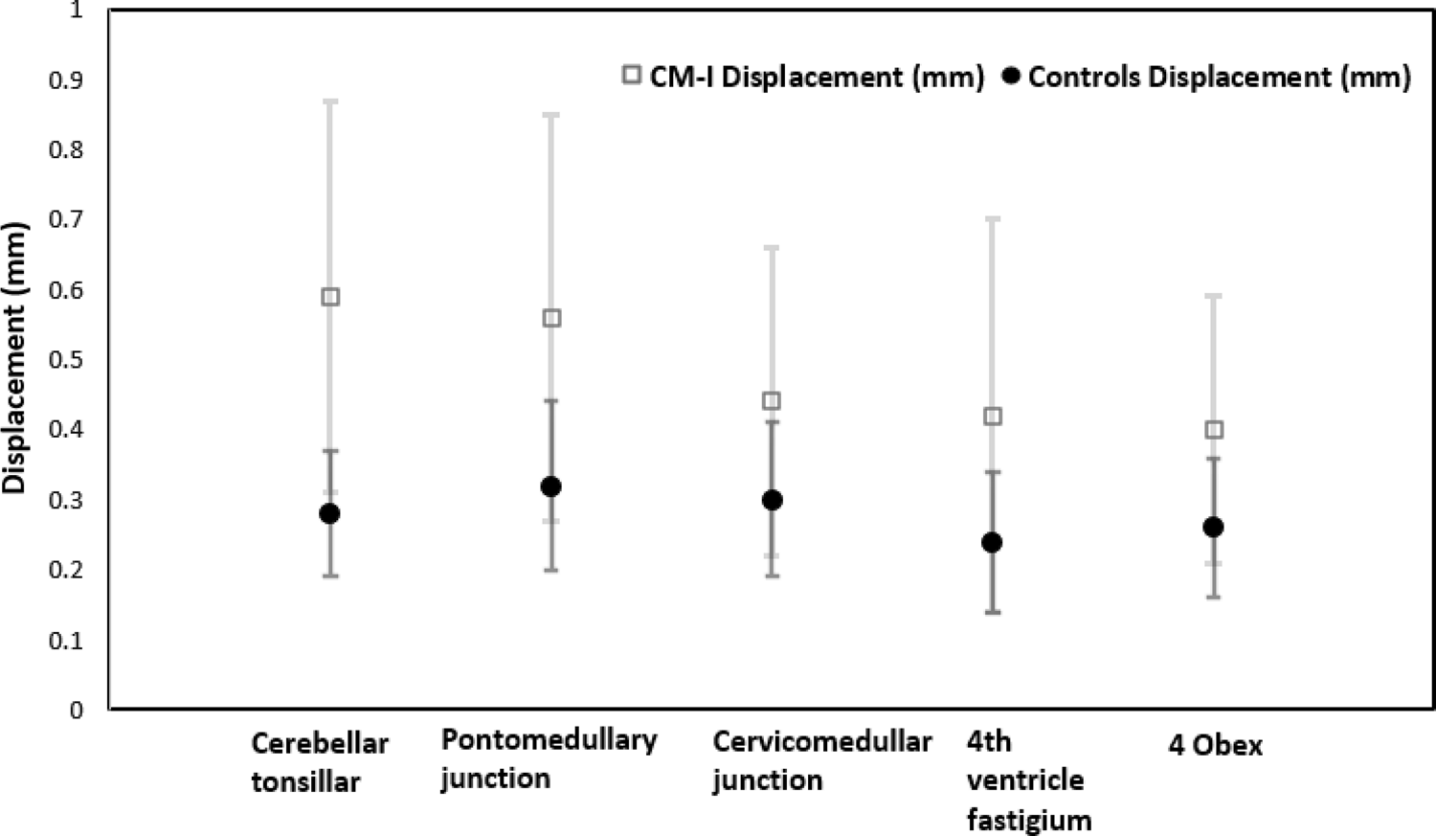
Tissue displacement estimates for differing brain regions of the brain in CM-I patients and controls (measured in sagittal section) [8, 27, 28]

Overall, three studies showed tonsillar motion is greater in CM-I patients compared to controls subjects (Fig. 5); these studies identified an increase in magnitude of downward caudal midbrain tissue displacement at the level of the brainstem and cranio-cervical junction in CM-1 patients [5]. This could be due to the additional pressure exerted by the CM on the spinal cord leading to an abrupt downward displacement of the spinal cord during systole, which impairs the return of CSF to the cranial cavity.

Table 2 compares data from CM-I patients and healthy controls. Larger BTPs are consistently observed in CM-I, especially in the cerebellum tonsillar region, where pooled analysis of data from CM-I patients and healthy participants (in 3 studies) suggested motion was 0.31 mm [95% CI: 0.23, 0.38, p < 0.0001] higher in CM-I patients compared to controls (Fig. 5) [8, 27, 28]. See Supplementary Appendix. 5 for more details of data included in this pooled analysis.

**Table 2:** A significant difference in mean values for cerebellar tonsillar region pulsations between CM-I patients and healthy controls was confirmed using an independent samples *t*-test [8, 27, 28]

Brain region	Mean ± SD of controls	Mean ± SD of CM-I	Difference in means
Cerebellar tonsillar (Sag)	0.28 ± 0.09, *n* = 31	0.59 ± 0.28, *n* = 75	0.31 [95% CI 0.23, 0.38]
Cerebellar tonsillar (Combined sag with axial of controls and compare it to sag only of CM-I)	0.31 ± 0.25, *n* = 41	0.59 ± 0.28, *n* = 75	0.28 [95% CI 0.17, 0.38]
Pontomedullary junction (Sag)	0.32 ± 0.12, *n* = 25	0.56 ± 0.29, *n* = 64	0.24 [95% CI 0.15, 0.32]
Cervicomedullary junction (Sag)	0.3 ± 0.11, *n* = 25	0.44 ± 0.22, *n* = 64	0.14 [95% CI 0.06, 0.21]
4th ventricle fastigium (Sag)	0.24 ± 0.1, *n* = 25	0.42 ± 0.28, *n* = 64	0.18 [95% CI 0.09, 0.26]
Obex (Sag)	0.26 ± 0.1, *n* = 25	0.4 ± 0.19, *n* = 64	0.14 [95% CI 0.07, 0.20]

CM-I studies confirmed that brain tissue motion is affected by the presence of disease, which can be investigated quantitatively using MRI cardiac-gated cine bSSFP sequences. Anatomical landmark motion tracking can then be used to measure displacements through the evaluation of pixel-shifting between frames. Such tracking of bSSFP images could provide an appropriate quantitative method to investigate brain motion in CM patients, due to its sensitivity to cephalad and caudad cerebellar tonsil motion. This method also achieves good SNR and is able to analyse contiguous blocks of data, however, standard cine bSSFP is not very sensitive to sub-voxel motion and therefore may not be as sensitive as DENSE, PC and aMRI to differences in BTP. The aMRI method also appears promising for visualising brain tissue motion in CM patients. Further studies are needed to develop aMRI for quantitative tissue motion measurements, which could potentially be achieved by using a phase-contrast MRI sequence for determining velocities and aMRI to amplify the tissue movements.

## Discussion

This is the first systematic review to provide an overview of MRI methods available for measuring brain tissue motion in humans. Following a prospective systematic search, 22 relevant records were retrieved, mainly feasibility studies.

There are 5 main methods used to investigate brain tissue motion, most of which use some form of cine cardiac-gating to acquire the images. Cardiac-gating used was either prospective or retrospective, triggered with pulse oximetry or ECG. Five main methods were used to measure motion in either one or all three spatial directions: (i) MRI utilising velocity encoding gradients for phase mapping to measure tissue velocity, (ii) CSPAMM or tagging sequence, (iii) DENSE to measure tissue displacement (iv) High-resolution anatomical MRI with retrospective analysis of “pixel-shifting” and tracking of anatomical landmarks to measure displacements and (v) aMRI.

Findings for whole and regional brain tissue motion from data available in reported healthy volunteer studies illustrated that peak brain tissue pulsations are found to be close to the brain stem, which decrease when propagating outwards to the peripheral brain lobes following ventricular systole.

Two studies found a correlation between the presence of tumour or IIH and decreased tissue displacement [17, 22]. Three studies found a correlation between the presence of CM-I and increased tissue displacement [5, 27, 28]. These studies appear to be the first to establish a link between structural pathology and BTP using MRI. This review also found a possible link between ICP and altered brain tissue motion, which suggests brain pulsations may decrease with increased ICP [22], which is also investigated by review work [33].

This MRI review found evidence to confirm that BTPs are affected by cerebral pathophysiology, which is also confirmed by ultrasound studies [2]. Changes in brain pulsatility due to disease pathophysiology were also established previously by Wagshul et al*.*, who discussed intracranial pulsatility using MRI and ultrasound modalities [33]. Each modality offers advantages and disadvantages and various approaches have been proposed by different research groups. For example, MRI has the advantage of enabling the direction of motion to be quantified and directly referenced to brain anatomy. However, the high cost of MRI scans, long scan durations, low temporal resolution and the need for large infrastructure are obvious disadvantages [34]. Doppler ultrasound offers better (real-time) temporal resolution than MRI, but anatomical imaging is poor or non-existent and measurements tend to be limited to a single beam-line or 2D plane. Ultrasound Doppler techniques are sensitive to the component of tissue motion in the direction of the ultrasound beam, but, with the exception of vector Doppler techniques, are unable to quantify both the magnitude and direction of tissue motion. Advantages and disadvantages of each modality was also discussed by Wagshul et al. [33].

MRI BTP amplitude estimates broadly agree with a previous ultrasound study by Turner et al*.* [12] who examined 107 healthy subjects and found a BTP amplitude of ~ 0.16 mm at a depth of 7.6 cm beneath the forehead, reducing to ~ 0.1 mm at a depth of 2.2 cm. Weaver et al*.* [18] examined 6 patients and found that BTP amplitude was approximately 0.15 mm close to the circle of Willis compared to 0.1 mm at the brain peripheries. It is reassuring that ultrasound and MRI measurements broadly concur, although, Figs. 2, 3, 4, suggest that BTP amplitude varies considerably for different brain regions and tissue structures, with the largest motion of ~ 0.24 mm being detected for the brainstem.

It appears from the 22 records of brain tissue motion studies in healthy subjects, all 5 MRI methods evaluated can be used for measuring BTP. On the other hand, for disease cases, BTP measurements have been limited to phase-contrast MRI, DENSE and anatomical landmark tracking with pixel-shifting. Moving forward from this, it would be advantageous to investigate whether other types of pathology have an impact on BTPs; including both acute and chronic conditions. It would also be useful to explore the suitability of different MRI methods for routine clinical use, with a view to obtaining short scan times and good SNR. Combining two methods, such as DENSE and aMRI, may also be useful for providing both qualitative and quantitative information.

The DENSE MR method appeared to be the most extensively used (8 out of 22 studies). The advantages of this method and ability to detect motion in the healthy and diseased brain have been replicated in multiple studies. Due to the exploratory nature of existing research and heterogeneity of studies combined with very small sample sizes, it was not appropriate to perform a meta-analysis; however, comparable displacement values in CM-I patients were pooled to summarise key findings.

It is remarkable that periodic displacements at the scale of microns can be detected using MR techniques. On this scale, noise and motion artefacts have potential to adversely impact measurements. Scans are performed over several minutes, during which time the subject’s head is likely to have moved by distances far greater than the sub-resolution distances being measured. The methods reviewed here address this in different ways. Compared with brain tissue motion, it can be assumed that the skull remains rigid and this could be used as a fiducial reference point.

Further studies should take care to systematically detail their methods and protocols to facilitate independent validation. Different, or unclear, methods and measurement parameters used between studies makes it difficult to directly compare results; a consensus statement may be useful to support pooling of data to prevent duplication of work. Reference data that systematically quantifies changes in pulsation estimates with participant age and physiological factors, such as blood pressure, may also prove useful when providing normative data on a population level for comparison with patient groups.

Despite heterogeneous techniques and reporting of different qualitative and quantitative measures, these studies do, however, support a broadly consistent picture of healthy brain tissue motion. Due to the potential of MRI to capture tissue motion in relation to anatomy, MRI is well suited to quantification of regional variations in displacement in the presence of focal pathology. However, clinical findings need to be reproduced on a larger scale than at present. Brain motion measurement algorithms have not yet been incorporated into clinical MR protocols and it remains to be seen whether MR measurement of brain tissue motion will prove clinically useful in the future.

## Limitations

The articles included in this review demonstrate that brain tissue movement can be measured using MRI; however, limiting our search to records published in English, means that some articles may have been overlooked. This review focused on records that measured natural brain pulsations occurring over the cardiac cycle. Inclusion of brain elastography literature reporting tissue strain and articles describing CSF dynamics [35], or brain motion due to respiration, may provide further insights but were omitted from our search criteria. Three studies excluded from this review used MRI for brain surface motion imaging (BSMI) [36–38] as a potential tool for assessing tumour–brain adhesion for surgical planning.

Since there are a small number of existing studies, it was difficult to make direct comparisons, or to form a strong opinion regarding clinical applicability. High heterogeneity between studies, small sample sizes and the absence of control subjects means a gold standard method for quantitatively measuring BTP still remains to be developed.

## Future work

As brain tissue pulsations are hypothesised to relate to cerebral blood flow, it is plausible that cerebrovascular pathology could result in altered brain tissue pulsatility [2]. A paucity of reference data exists for healthy participants. Sample sizes in existing studies were typically small (1–25 subjects). Further work, examining a larger number of healthy subjects, may be valuable in establishing variability of brain tissue motion for sample size estimation and to provide further reference data for clinical comparison.

Additional MRI investigations will be required to determine how specific pathologies may affect motion in different brain regions. To date, no researchers have used MRI to study the impact of stroke or head trauma on brain tissue motion. The impact of physiological processes and CSF changes relating to sleep, respiration, cognitive changes and cerebrovascular physiology on BTPs also remains poorly understood. Future studies investigating factors that could affect brain tissue motion are needed. Finally, unifying reported measures and standardisation of techniques could assist in advancing the field and preventing duplication of work.

A further application of BTP measurements might be found in supporting motion corrections for high-resolution MR. This was not mentioned or discussed in any of the included papers, although concerns around blurring of MRI images did prompt some early studies [6, 16]. With MR technology moving towards higher resolution imaging, as well as informing high precision therapeutic treatments (e.g. stereotactic radiotherapy), there may be a growing interest in using MR-based BTP measurements to support motion correction [39]. Finally, no studies have so far considered cardio-ballistic effects [40].

## Conclusion

An increasing body of research has now investigated the use of different MRI techniques to study the mechanical properties and motion of the human brain in vivo. This systematic review identified 22 records assessing brain tissue motion. All methods successfully either visualised and/or quantified natural BTPs in humans using MRI. The available evidence suggests that brain tissue motion differs between brain regions, with central brain regions experiencing the strongest pulsations followed by the motion of peripheral regions. Studies in patients with tumours, IIH and CM-I malformation show that BTPs can be affected by neuropathological disorders.

As BTPs are influenced by both haemodynamic and mechanical factors, a better understanding of BTPs may be of scientific value in understanding the relationships between blood, brain tissue and CSF in both health and disease. Based on this review, we conclude that MR techniques provide accurate quantitative and qualitative information about regional tissue motion and can be used to detect changes in motion associated with pathology. This knowledge may make a valuable scientific contribution towards our understanding of the brain, although clinical applications have yet to emerge.

Although MRI provides a method of measuring brain motion, typical values for a general healthy population are not yet available. Further healthy participant data should be collected to act as healthy subject reference data. Areas of future interest could include the investigation of brain motion in a range of physiological and pathophysiological conditions to inform the development of models and hypotheses linking brain biomechanics and physiology to BTP observations.

## Supplementary Information

Below is the link to the electronic supplementary material.

Supplementary file1 (DOCX 304 KB)
