# Supplementary material for: Magnetic resonance imaging of the pulsing brain: a systematic review
Source: MAGMA. 2022 Oct 15;36(1):3–14. doi: 10.1007/s10334-022-01043-1 (PMC9992013; doi:10.1007/s10334-022-01043-1)
Supplement: Supplementary file 1 — Supplementary file1 (DOCX 304 KB) [file 10334_2022_1043_MOESM1_ESM.docx]

**Appendix 1** Summary of retrieved records examining brain tissue motion using MRI techniques

| Author, Year | Study Design | Population (m= male, f=female) | Age Range (years) | Type of sequence and acquisition | Imaging planes (S=sagittal, C=coronal, A=axial) | BTP measurement results |
| --- | --- | --- | --- | --- | --- | --- |
| Feinberg *et al.,* 1987 | Prospective cohort | 25 healthy adults | 15-43 | Cardiac-gated spin echo 2D Fourier Transform imaging method utilizing phase incremental velocity encoding gradients for direct velocity measurements | 2D  S, C, A | Maximum velocities occurred in the brain stem (1.3 mm/s). |
| Greitz *et al*., 1992 | Prospective feasibility | 15 healthy adults (14 m, 1 f) | 20- 44 | Cardiac-gated standard spin echo phase contrast sequence with pair of unipolar velocity encoding gradients | 2D  S, C, A | Maximum velocities occurred in the brain stem (1.5 mm/s) and basal ganglia (1.0 mm/s). |
| Poncelet *et al.*, 1992 | Prospective feasibility | 14 healthy adults (9 m, 5 f) | 20- 40 | Cine ECG gated spin echo phase contrast MR sequence with EPI readout and a pair of unipolar velocity encoding gradients | 2D  C, A | Maximum velocities occurred in the brain stem (2 mm/s) and thalami (1.5 mm/s). |
| Enzmann *et al.,* 1992 | Prospective feasibility | 10 healthy adults (6 m, 4 f) | Male: 26-36  Female: 23-34 | Cardiac-gated cine standard gradient echo phase contrast MR sequence with pair of bi-polar velocity encoding gradients | 2D  S, C, A | Peak brain displacement was in the range of 0.1-0.5 mm for all brain structures except the cerebellar tonsils, which had greater displacement (0.4 mm). |
| Wirestam *et al.,* 1997 | Retrospective feasibility | 8 healthy adults (5m, 3 f)  3 brain tumour patients; 2 male patients with astrocytoma (grade I-II and III-IV), and one female patient with meningioma | Healthy:  Male: 23 – 41 Female: 25-28  Patients:  Male: 36 and 73  Female: 65 | ECG gated standard gradient-echo pulse sequence with bi-polar velocity encoding gradients | 2D  A | In healthy, maximum velocities were found in the central parts of the brain (thalamus) of 1.5 mm/s with weak or no motion in frontal lobe.  In patients with astrocytoma, the maximum velocities in the central parts of the brain were 1.1 in I-II and 0.7 mm/s in III-IV.  In patient with meningioma, the velocities in the central parts of the brain were generally low (0.3 mm/s). |
| Soellinger *et al.,* 2007 | Prospective feasibility | 10 healthy adults (7 m, 3f) | Mean age: 27 (range= 22–61) | Cine CSPAMM tagging sequence with harmonic phase (HARP) post processing | Not mentioned  S | Peak displacement was in the pons (0.18 mm), which decreased cranially to 0.052 mm in the corpus callosum, and to 0.041 mm in the frontal lobe. |
| Soellinger *et al.*, 2009 | Prospective feasibility | 12 healthy adults (5 m, 7 f) | Mean age: 26.3 | ECG gated cine DENSE sequence with EPI readout and displacement encoding gradients. | 3D  S, C, A | Peak displacements were in the central brain regions. (FH): thalamus (0.13 mm), (RL): thalamus (0.06 mm), and (AP): caudate nucleus (0.05 mm). |
| Zhong *et al.,* 2009 | Prospective feasibility | 3 healthy adults (male) | 29- 42 | ECG gated cine DENSE spiral sequence with unipolar motion encoding gradient | 2D  S, C, A | Peak displacement less than 0.3 mm. Eulerian 2D displacement vectors were determined in the imaging plane. |
| Cousins *et el.,* 2009 | Retrospective case control | 17 adults; 11 CM-I patients (4 m, 7 f) vs. 6 non-CM-I controls (sex not stated) | Patients: 29–50  Controls: 20–50 | Cardiac-gated cine using a Fast imaging employing steady-state acquisition (FIESTA) sequence | 2D  S | The average total motion was 0.43 mm in controls and 0.57 mm in patients with CM-I, 33%. |
| Weaver *et al.,* 2012 | Retrospective feasibility | 6 healthy adults (4 m, 2 f) | 25 – 55 | Retrospectively gated gradient echo phase contrast sequence with pair of bipolar velocity encoding gradients | 2D  A | Most of the tissue displacement occurred around the Circle of Willis (mean= 22 mm). |
| Zorgani *al el.,* 2015 | Prospective feasibility | 2 healthy adults (sex not mentioned) | Not mentioned | Gradient echo phase encoding MR sequence with single shot EPI readout and a pair of bipolar motion encoding gradients | 2D  S, A | Motion amplitudes of whole brain tissue are relatively small at only 5 μm. |
| Leung *et al.,* 2016 | Retrospective case control | 89 adults; 64 adult CM-I patients (‘mostly male’)  25 adult Controls | CM-I: mean age 32.7  Controls: mean age 32.0 | Cardiac-gated (ECG or pulse oximetry triggered) cine balanced fast-field echo (FFE) sequence | 2D  S | CM-I patients had greater cerebellar tonsillar motion in both the AP and SI directions than controls (AP= +0.34 mm, SI= +0.49 mm). |
| Holdsworth *et al.,* 2016 | Retrospective  feasibility | 4 healthy adults (2 m, 2 f) | 29- 64 | Amplified MRI (aMRI), utilizing a cardiac-gated cine balanced steady-state free precession (bSSFP) sequence and Eulerian video magnification  (EVM) | 2D  S | Brain displacement occurred especially in brainstem, cerebellum, and spinal cord. |
| Terem *et al.,* 2018 | Retrospective feasibility | 1 CM-I paediatric patients and 1 control (2 m) | Control: 3  CM-I : 4 | Amplified MRI (aMRI), utilizing a cardiac-gated cine balanced steady-state free precession (bSSFP) sequence, with a phase-based motion amplification algorithm | 2D  S | Brain tissue displacements at the level of the brainstem and craniocervical junction were greater in CM-I patient compared to controls. |
| Pahlavian *et al.,* 2018 | Prospective feasibility | 8 healthy adults (7 m, 1 f) | Mean age: 28 | ECG gated cine DENSE sequence with Spiral readout and displacement encoding gradients. | 2D  S, C | Peak displacement was measured in the brain stem (187 µm) followed by the cerebellum (105 µm). Smaller brain tissue displacements were in more peripheral structures. |
| Saindane *et al.,* 2018 | Prospective feasibility | 9 IIH patients (9 f) vs 9 healthy control subjects (7 m, 2 f) | Patients: median age 28.1  Healthy: median age 34.0 | Cardiac-gated cine DENSE with EPI sequence | Not mentioned  A, S | Patients with IIH had lower brain pontine motion (0.060 mm) compared to control subjects (0.109 mm). |
| Sloots *et al.,* 2018 | Prospective feasibility | 7 healthy subjects (3 f, 4 m) | Average age: 25 | Cardiac-gated cine DENSE 2D EPI sequence | 2D  A, S | Displacement was largest in the deep brain (basal ganglia) and was on average from -99±30µm to -139±17µm. |
| Adams *et al.,*  2019 | Prospective feasibility | 8 healthy adults (3 m, 5 f) | Average age: 24 | Cardiac-gated 3D cine DENSE sequence with displacement encoding gradients and EPI readout | 3D  A, S, C | Mean peak whole brain volumetric strain at 7 T was (4.5 ± 1.0) × 10−4 and at 3 T was (5.1 ± 1.2) × 10−4.  The peak volumetric strain ratio of grey to white matter was 4.4 ± 2.8 at 7 T and 4.0 ± 1.2 at 3 T. |
| Adams *et al.,*  2019 | Prospective feasibility | 8 healthy adults (3 f, 5 m) | Mean age: 27 | Cardiac-gated cine DENSE 3D EPI sequence with motion encoding gradients along AP, RL and FH directions. | 3D  Not mentioned | First/second session to measure peak volumetric strains:  For whole brain were: (6.4 ± 17) x10^-4^ / (6.7 ± 1.6) x10^-4^.  For white matter were: (9.5 ± 2.5) x10^-4^/ (9.6 _ 2.4) x10^-4^, and for grey matter, and (4.4 ± 1.7) x 10^-4^ / (4.1 ± 0.8) x10^-4^. |
| Sloots *et al.,* 2020 | Prospective feasibility | 9 healthy volunteers (6 m, 3 f( | Average age:29 | Cardiac-gated cine DENSE 2D EPI sequence | 2 D  S, C | Peak volumetric strains were in the basal ganglia with median of 0.85x10^-3^  Smaller volumetric strains were observed in the white matter range from 0.28x 10^-3^ to 0–0.59 x10^-3^. |
| Abderezaei *et al.,* 2021 | Prospective feasibility | 6 healthy adults (2 f, 4 m) | 26 - 36 | Amplified MRI (aMRI) in silico, utilizing a cardiac-gated cine balanced steady-state free precession (bSSFP) sequence | 3 D  A, S, C | Demonstrated a substantial motion near the pons and midbrain in which approximately 76% ± 6% of the motion was towards the superior-inferior and anterior-posterior direction, whereas, about 20% ± 10% was towards the medial-lateral direction. |
| Terem *et al.,* 2021 | Prospective feasibility | 2 healthy volunteers (1 m, 1 f( | 25 and 65 | Amplified MRI (aMRI), utilizing a cardiac-gated cine balanced steady-state free precession (bSSFP) sequence. | 3 D  A, S, C | Displacement was in the cranial- caudal direction in the sagittal and coronal planes, and expanding motion in the axial plane, with the largest brain tissue displacement occurring around the midbrain, cerebellar tonsils, brainstem, and hypothalamus.  Minimal displacement occurred in the frontal lobe, parietal lobe, occipital lobe, temporal lobe, and posterior cerebellum. |

**Appendix 2** The pre-agreed quality assessment tool


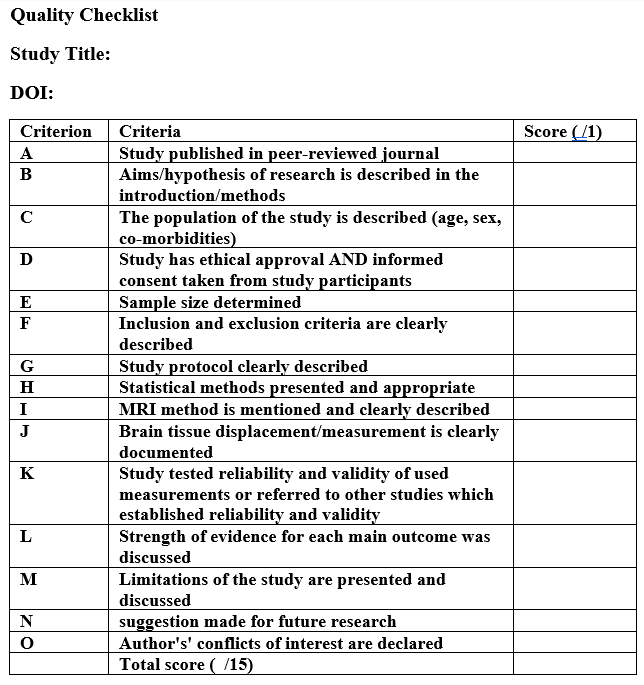


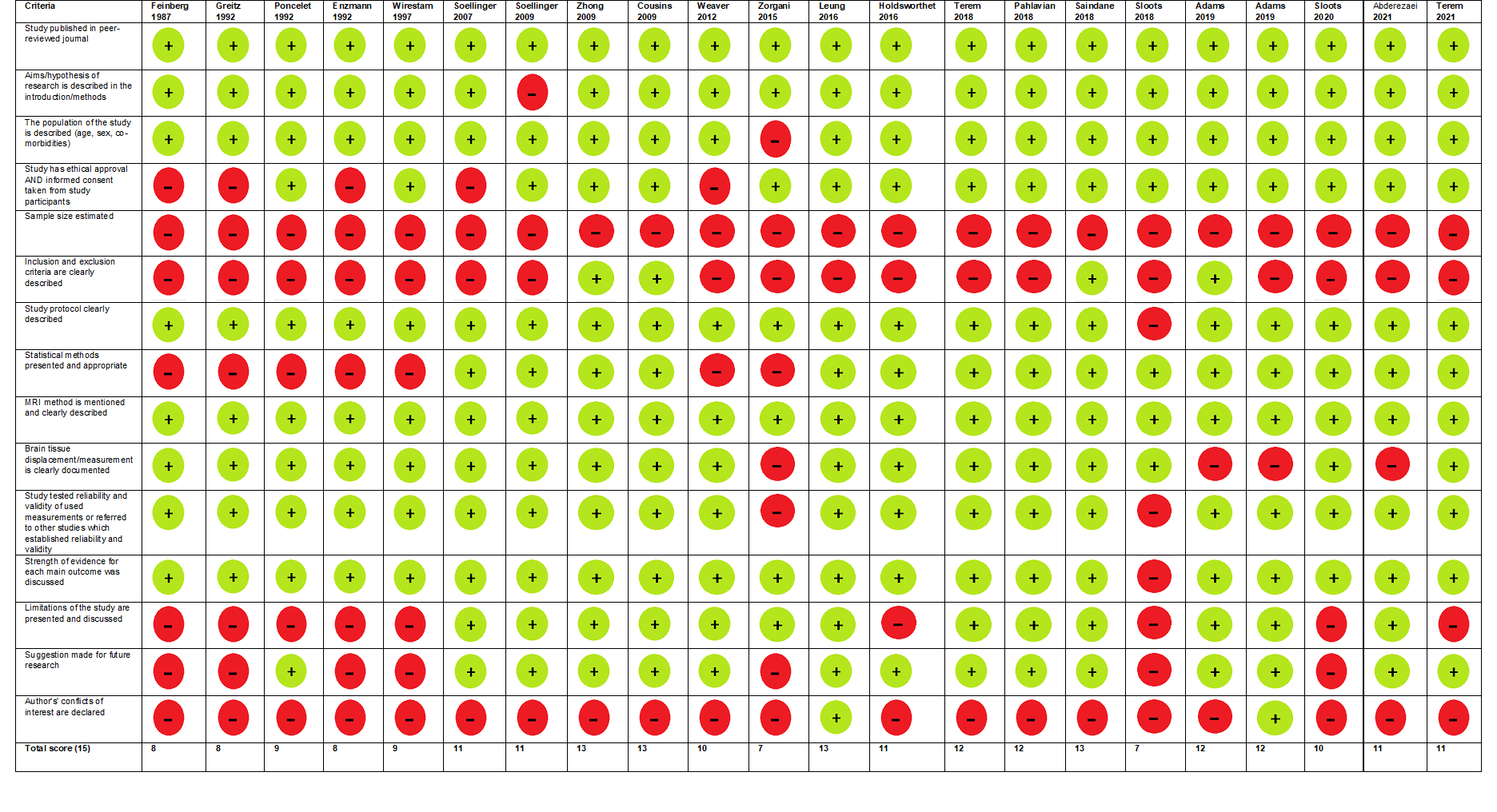


**Appendix 3** Risk of bias summary for the 22 studies.

**Appendix 4** Tissue maximum velocity estimates for differing brain regions in 6 studies

| Max velocity (mm/sec) | Tissue RoI | Imaging plane | Sequence | References |
| --- | --- | --- | --- | --- |
| 0.4 | Anterior cerebral  cortex (n=3) | Sagittal and axial (n=24) | Cardiac gated spin echo phase with a pair of equal velocity encoding gradient pulses for 2D Fourier transform (2DFI) Mean velocity measured at different phases of the cardiac cycle. Maximum of the mean velocities determined.  Total n= 25 healthy subjects | Feinberg *et al.* |
| 0.4 | Corpus callosum (n=4) |  |  |  |
| 1.3 | Brain Stem (n=5) |  |  |  |
| 0.63 | Foramen of Monno (n=12) |  |  |  |
| 1 (range 0.6-1.7) | Basal Ganglia (n=12) | Axial (n=12) | Cardiac gated standard spin echo phase contrast sequence with pair of unipolar velocity encoding gradients.  Mean velocity measured at different phases of the cardiac cycle. Maximum of the mean velocities determined.  Total n= 15 healthy subjects | Greitz *et al.* |
| 1.5 (range 1.1-2.1) | Pons (n=5) |  |  |  |
| 0.5 bilaterally | Basal Ganglia | Sagittal (n=1) |  |  |
| 0.3 | Posteromedial thalamus | Coronal (n=3) |  |  |
| 1.1 | Vermis |  |  |  |
| 1.5 | Pons |  |  |  |
| 2.3 | Medulla oblongata |  |  |  |
| 0.29, 0.07, 0.09 | Frontal lobe | Axial (Velocities represent in cephalocaudal n= 6 subjects, mediolateral n= 3 subjects, and anteroposterior n= 2 subjects, retrospectively) | Cine ECG gated spin echo EPI phase contrast sequence with pair of unipolar velocity encoding gradients.  Mean velocity measured at different phases of the cardiac cycle. Maximum of the mean velocities determined.  Total n = 14 healthy subjects | Poncelet *et al.* |
| 0.26, 0.11, 0.12 | Occipital lobe |  |  |  |
| 0.91, 0.59, 0.33 | Deep grey matter |  |  |  |
| 0.49, 0.16, 0.17 | Corpus callosum, anterior |  |  |  |
| 0.36, 0.22, 0.15 | Corpus callosum, posterior |  |  |  |
| 2 | Brain stem (n=1 subject) | Coronal |  |  |
| 1.5 | Thalami (n=1 subject) |  |  |  |
| 1.5 | Brain stem – medulla | Axial (n=10) | Cine standard gradient echo phase contrast sequence with pair of bipolar velocity encoding gradients. Mean velocity measured at different phases of the cardiac cycle. Maximum of the mean velocities determined.  Total n = 10 healthy subjects | Enzmann *et al.* |
| 4.8 | Cerebellar tonsil |  |  |  |
| 1.2 | Occipital lobe |  |  |  |
| 1.1 | Frontal lobe | Sagittal (n=10) |  |  |
| 1.2 | Posterior cerebellum |  |  |  |
| 1.1 | Hypothalamic | Coronal (n=10) |  |  |
| 0.65 | Parietal lobe |  |  |  |
| 1.5 | Thalamus | Axial (n=6) | ECG gated standard gradient-echo pulse sequence with bi-polar velocity encoding gradients. Mean velocity measured at different phases of the cardiac cycle. Maximum of the mean velocities determined.  Total n = 8 healthy subjects | Wirestam *et al.* |
| ~3.20 cm/s (~32 mm/s) | Cerebellar tonsillar | Sagittal (n= 25 controls) | Cardiac-gated (ECG or pulse oximetry triggered) cine balanced fast-field echo (FFE) sequence.  Motion tracking of anatomical markers. Velocity measured using consecutive  magnitudes of displacement  (velocity = displacement/  time).  Total n= 89 participants | Leung *et al.* |

**Appendix 5** Details of data pooled in 3 studies to estimate tissue displacement for differing brain regions of the brain in CM-I patients and controls.

| Study reference | RoI | Section planes | Number of subjects | Estimate motion (mm) | SD (mm) | Mean-weighted average (mm) | SD-weighted average (mm) |
| --- | --- | --- | --- | --- | --- | --- | --- |
| Enzmann *et al.*, 1992 | Cerebellar tonsillar of controls | Axial | 10 | 0.4 | 0.5 | Cerebellar tonsillar of controls = 0.31 | Cerebellar tonsillar of controls = 0.25 |
| Cousins *et al.*, 2009 | Cerebellar tonsillar of controls | Sag | 6 | 0.43 | 0.06 |  |  |
| Leung *et al.*, 2016 | Cerebellar tonsillar of controls | Sag | 25 | 0.25 | 0.10 |  |  |
| Cousins *et al.*, 2009 | Cerebellar tonsillar of CM-I | Sag | 11 | 0.57 | 0.04 | Cerebellar tonsillar of CM-I = 0.59 | Cerebellar tonsillar of CM-I = 0.28 |
| Leung *et al.*, 2016 | Cerebellar tonsillar of CM-I | Sag | 64 | 0.59 | 0.30 |  |  |
| Leung *et al.*, 2016 | Obex of CM-1 | Sag | 64 | 0.40 | 0.19 |  |  |
| Leung *et al.*, 2016 | 4th ventricle fastigium of CM-1 | Sag | 64 | 0.42 | 0.28 |  |  |
| Leung *et al.*, 2016 | Pontomedullary junction of CM-1 | Sag | 64 | 0.56 | 0.29 |  |  |
| Leung *et al.*, 2016 | Cervicomedullary junction of CM-1 | Sag | 64 | 0.44 | 0.22 |  |  |
| Leung *et al.*, 2016 | Obex of controls | Sag | 25 | 0.26 | 0.10 |  |  |
| Leung *et al.*, 2016 | 4th ventricle fastigium of controls | Sag | 25 | 0.24 | 0.10 |  |  |
| Leung *et al.*, 2016 | Pontomedullary junction of controls | Sag | 25 | 0.32 | 0.12 |  |  |
| Leung *et al.*, 2016 | Cervicomedullary junction of controls | Sag | 25 | 0.30 | 0.11 |  |  |
| Leung *et al.*, 2016 | Obex of controls | Sag | 25 | 0.26 | 0.10 |  |  |
